# Bone reporting and data system on CT (Bone-RADS-CT): a validation study by four readers on 328 cases from three local and two public databases

**DOI:** 10.1186/s13244-025-02057-8

**Published:** 2025-08-12

**Authors:** Yue Xing, Defang Ding, Shun Dai, Yangfan Hu, Xianwei Liu, Liangjing Lyu, Guangcheng Zhang, Shiqi Mao, Qian Yin, Junjie Lu, Jiarui Yang, Yang Song, Huan Zhang, Chengzhou Li, Weiwu Yao, Jingyu Zhong

**Affiliations:** 1 Department of Imaging, Tongren Hospital, Shanghai Jiao Tong University School of Medicine, Shanghai, China; 2 Shanghai Key Laboratory of Flexible Medical Robotics, Tongren Hospital, Institute of Medical Robotics, Shanghai Jiao Tong University, Shanghai, China; 3 Department of Orthopedics, Shanghai Sixth People’s Hospital, Shanghai Jiao Tong University School of Medicine, Shanghai, China; 4 Department of Medical Oncology, Shanghai Pulmonary Hospital, Tongji University School of Medicine, Shanghai, China; 5 Department of Pathology, Renhe Hospital, Shanghai, China; 6 Department of Epidemiology and Population Health, Stanford University School of Medicine, Stanford, California USA; 7 Department of Biomedical Engineering, Boston University, Boston, Massachusetts USA; 8 MR Research Collaboration Team, Siemens Healthineers Ltd., Shanghai, China; 9 Department of Radiology, Ruijin Hospital, Shanghai Jiao Tong University School of Medicine, Shanghai, China; 10 Department of Nuclear Medicine, Tongren Hospital, Shanghai Jiao Tong University School of Medicine, Shanghai, China

**Keywords:** Bone neoplasms, Clinical decision-making, Reproducibility of results, Multidetector computed tomography

## Abstract

**Objective:**

To evaluate the reproducibility and effectiveness of the bone reporting and data system on CT (Bone‐RADS-CT) for incidental solitary bone lesions in adults.

**Materials and methods:**

We retrospectively included 328 CT cases from three local and two public databases, respectively. All the cases were histopathologically or clinically confirmed bone lesions, “do not touch” lesions with typical appearance, and remained stable for at least 2 years. Each lesion with gender, age, and clinical history was categorized according to the Bone-RADS algorithm by two musculoskeletal radiologists and two non-musculoskeletal radiologists. The Bone-RADS categories were as follows: Bone-RADS-1, likely benign, leave alone; Bone-RADS-2, incomplete assessed on imaging, perform different imaging modality; Bone-RADS-3, intermediate, perform follow-up imaging; Bone-RADS-4, suspicious for malignancy or need for treatment, biopsy and/or oncologic referral. Inter-reader agreement was evaluated. The diagnostic performance of the Bone-RADS-CT for distinguishing positive cases (intermediate or malignant lesions or osteomyelitis) from negative cases (benign lesions), were measured, using histopathology results, clinical diagnosis, or follow-up as a standard reference.

**Results:**

There were 223 positive cases and 105 negative cases, respectively. The overall inter-reader agreement between two musculoskeletal and two non-musculoskeletal radiologists were both moderate (weighted kappa 0.553 and 0.403). The diagnostic performance for identifying intermediate or malignant lesions or osteomyelitis ranged according to radiologists with sensitivities of 88.8% to 94.6%, specificities of 42.9% to 71.1%, and accuracies of 78.0% to 86.6%.

**Conclusion:**

Bone-RADS-CT is effective for identifying bone lesions that need further treatment, but is only moderately reliable for readers of different specialties and experience.

**Critical relevance statement:**

Bone-RADS-CT has been demonstrated to be a reliable algorithm for non-musculoskeletal radiologists and an effective tool for identifying the “need for treatment” incidental solitary bone lesions in adults, but still needs improvement in the rating method and category definition.

**Key Points:**

- Bone-RADS-CT has been demonstrated to be reliable and accurate when rated by musculoskeletal radiologists.
- Bone-RADS-CT achieved moderate agreement for musculoskeletal and non-musculoskeletal radiologists.
- Bone-RADS-CT presented high sensitivities but low specificities for identifying “need for treatment” bone lesions.

**Graphical Abstract:**

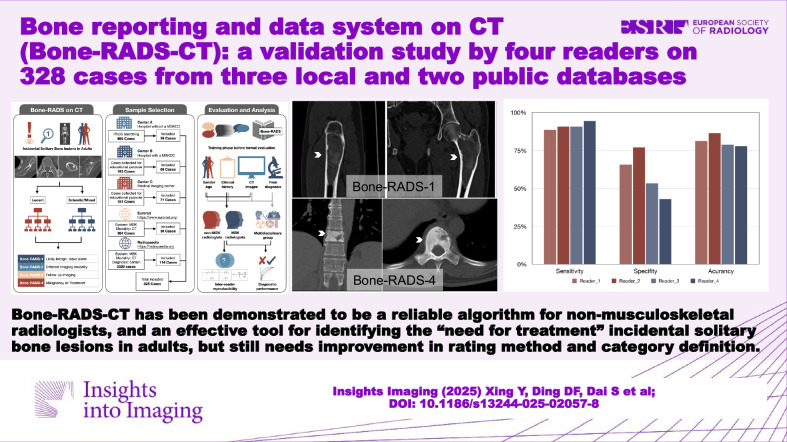

## Introduction

An increasing number of bone lesions are detected for unrelated reasons with the higher availability and lower cost of imaging examinations [1]. The diagnosis of bone lesions can sometimes be difficult even for musculoskeletal radiologists, as the differential diagnostic spectrum includes tumors from benign, intermediate, to malignant, as well as infection, inflammation, infarction, and bone involvement of systemic diseases [2–6]. The relative rarity of primary bone malignancies means a limited number of cases [7–9], which may lead to insufficient expertise of radiologists to identify and evaluate bone lesions. The overlapping imaging features of benign and malignant lesions bring extra difficulties in evaluation, and have a negative impact on the inter-reader reliability and diagnostic performance [2–6]. Owing to these difficulties, the diagnosis of bone lesions would be delayed, and the suboptimal management could potentially lead to poor prognosis [10, 11]. Thus, a systematic and standardized approach for bone tumor evaluation is required [12, 13]

A series of reporting and data system has been proposed for many system areas, and enhances the communication and collaboration among multiple clinical specialties by having a shared concept on lesions [14, 15]. There are also several systems for bone lesions [16]. Radiological evaluation score for bone tumors (REST) [17] and bone tumor risk stratification and management system developed by the American College of Radiology (ACR Bone-RADS) [18] are two systems that were developed for bone lesion evaluation on radiographs. The ACR Bone-RADS has already shown high sensitivity and moderate inter-reader agreement, but relatively low specificity [19, 20]. Osseous tumor reporting and data system (OT-RADS) [21, 22] is a system based on conventional MRI with or without diffusion weighted imaging sequence. Bone tumor imaging reporting and data system (BTI-RADS) [23] relies on both CT and contrast-enhanced MRI for assessment. The REST is interpreted in a binary manner [17], while the other systems are reported in four to six grades with increasing possibility of malignancy [18, 21–23]. The latest bone reporting and data system developed by the Society of Skeletal Radiology (SSR Bone-RADS) [24] differs from the ACR Bone-RADS and is applicable to either CT or MRI. The Bone-RADS categories are generated mainly regarding the later management and are expected to be more practical in clinical routine. The Bone-RADS categories are Bone-RADS-1, likely benign, leave alone; Bone-RADS-2, incomplete assessed on imaging, perform different imaging modality; Bone-RADS-3, intermediate, perform follow-up imaging; Bone-RADS-4, suspicious for malignancy or need for treatment, biopsy and/or oncologic referral. The Bone-RADS has been validated and has shown good diagnostic performance with moderate inter- and intra-reader reliability [25, 26]. A revised version of Bone-RADS further improved its specificity and maintained the sensitivity [27]. However, these studies were performed within a single center and were mainly conducted with experienced musculoskeletal radiologists. As Bone-RADS may be more commonly used outside of a musculoskeletal practice, the validation among non-musculoskeletal radiologists is necessary. Furthermore, to test the algorithm across a wider range of disease spectra, we decided to conduct a multicenter study using both local and public data sources.

Therefore, this study aimed to evaluate the reproducibility and effectiveness of the Bone‐RADS-CT for incidental solitary bone lesions in adults by two musculoskeletal radiologists and two non-musculoskeletal radiologists using three local databases and two public databases.

## Materials and methods

### Study design

**Fig. 1:**
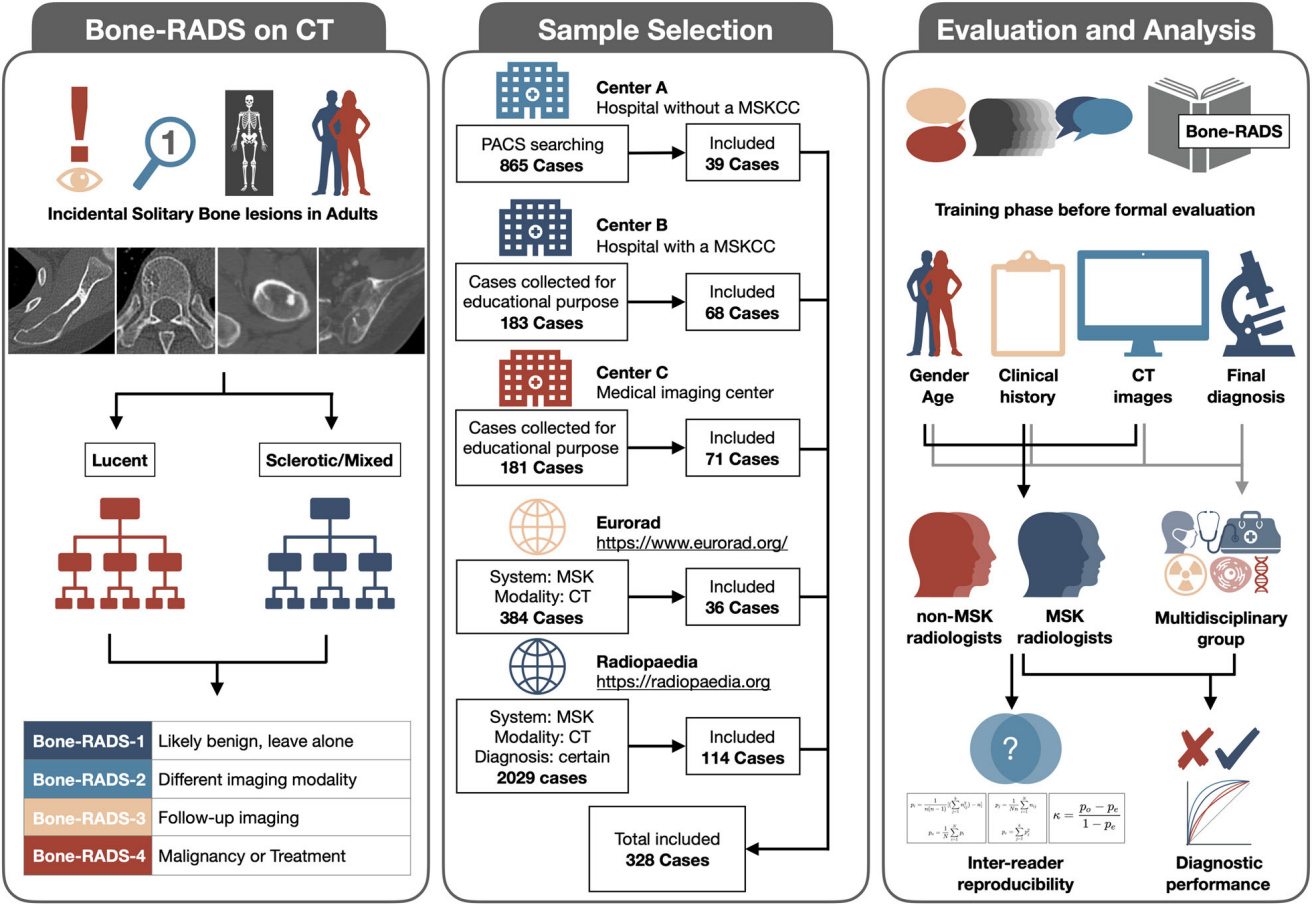
Bone-RADS-CT and workflow of the study. Bone-RADS, bone reporting and data system; MSKCC, musculoskeletal cancer center; PACS, picture archiving and communication system

This is a retrospective study using cases from three local institutional databases and two public databases (Fig. 1). This study has received institutional ethics approval (2022-029-01). Written informed consent from participants was waived. The two public databases are Eurorad (https://www.eurorad.org) and Radiopaedia (https://radiopaedia.org). We have discussed these two databases with the editorial team and were permitted to use the cases for research purposes under the Creative Commons License CC BY-NC-SA 4.0 and CC BY-NC-SA 3.0, respectively.

### Study sample selection

Two musculoskeletal radiologists with 6 and 7 years of experience (J.Y.Z. and Y.X.) selected the cases for investigation [17, 19–23, 25–27]. The inclusion criteria were: (1) ≥ 18 years old; (2) solitary bone lesions; (3) complete CT examination with a slice thickness of at least 5 mm, and appropriate window width and level for bone and soft tissue evaluation; (4) clinical history available; and (5) histologically or clinically confirmed diagnosis, those with typical appearance of “do not touch” lesions and remain stable for at least 2 years. The exclusion criteria were: (1) overlapping cases, (2) follow-up examinations, (3) examinations after treatment, (4) unsatisfactory image quality, and (5) allowed for non-commercial use. The sample selection process is described in detail (Supplementary Note S1).

Three local institutional databases and two public databases were screened. For center A, the picture archiving and communication system was searched using terms related to bone lesions, and 865 potential cases between 01 Jan 2019 and 30 Jun 2023 were assessed. For center B, 183 potential cases with histological results between 01 Jan 2018 and 30 Jun 2020 were collected for educational purposes and evaluated. For center C, 181 potential cases with histological results between 01 Jan 2018 and 30 Jun 2020 were collected for research purposes and evaluated. For Eurorad, 384 potential cases until 30 Jun 2024 were identified with the restriction of the musculoskeletal system and imaging modality of CT, and then assessed. For Radiopaedia, 2741 potential cases until 30 Jun 2024 were identified with the restriction of musculoskeletal system, imaging modality of CT, and certain diagnoses, and then assessed. Finally, 39, 68, 71, 36, and 114 cases from center A, center B, center C, Eurorad, and Radiopeadia databases were included, respectively, resulting in 328 cases in total. Our post hoc sample size calculation indicated that the sample size is appropriate for the current study (Supplementary Note S2) [28].

### Final diagnosis and Bone-RADS

Our multidisciplinary group determined the final diagnosis of cases and reference standard of Bone-RADS for diagnostic performance evaluation (Supplementary Note S3). Our group consisted of six radiologists (J.Y.Z., Y.X., C.Z.L., L.J.L., H.Z., and W.W.Y.), one orthopedist (G.C.Z.), one oncologist (S.Q.M.), and one pathologist (Q.Y.). The final diagnosis and Bone-RADS category were reached according to the histological or clinical confirmation, typical appearance of “do not touch” lesions and follow-ups for at least 2 years [17, 19–23, 25–27]. For diagnostic performance evaluation, the intermediate or malignant lesions or osteomyelitis were defined as positive cases, and benign lesions were defined as negative cases. The cases were calculated as follows: Bone-RADS-4 cases that diagnosed as Bone-RADS-2, 3, or 4, true positive (TP); Bone-RADS-1 cases that diagnosed as Bone-RADS-2, 3, or 4, false positive (FP); Bone-RADS-4 cases that diagnosed as Bone-RADS-1, false negative (FN); and, Bone-RADS-1 cases that diagnosed as Bone-RADS-1, true negative (TN). We used these definitions to tell whether the lesions designated Bone-RADS-1 were truly benign processes that require no additional work-up, while the Bone-RADS-4 lesions were suspicious for malignancy or need for treatment.

### Bone-RADS training and evaluation

The Bone-RADS category was rated for each case by two musculoskeletal radiologists with 6 and 8 years of experience (Y.F.H. and D.F.D.), and two non-musculoskeletal radiologists, both with 9 years of experience (S.D. and X.W.L.), respectively (Supplementary Note S4). Before the formal assessment, the readers read the Bone-RADS white paper, tested themselves using ten attached representative cases, and discussed with the multidisciplinary group until they had a shared comprehension of Bone-RADS [24]. For the formal assessment, two musculoskeletal radiologists with 6 and 7 years of experience (J.Y.Z. and Y.X.) created anonymized image sets in random order using the PowerPoint software (Office 365; Microsoft) and summarized their gender, age, and clinical history using Excel software (Office 365; Microsoft). The readers were asked to rate the Bone-RADS category for each case according to the PowerPoint and Excel documents, with blindness to the data source and final diagnosis. There was no restriction on reading time for each case, but all the readers finished the Bone-RADS rating within a week. This method was considered to be effective for the validation of an RADS in the musculoskeletal system [29].

### Statistical analysis

The statistical analysis was performed using R language (version 4.1.3; https://www.r-project.org) within RStduio software (version 1.4.1106; https://posit.co). The continuous and categorical variables were presented as mean ± standard deviation and distribution (percentage), respectively. For the differences among the five databases, one-way analysis of variance was used for continuous variables, while the chi-square test or Fisher’s exact test was applied for categorical variables. For inter-reader agreement, weighted kappa statistics was estimated, and interpreted as follows: poor, < 0.20; fair, 0.20–0.40; moderate. 0.40–0.60; good, 0.60–0.80; and excellent, ≥ 0.80 [30]. For diagnostic performance, sensitivities, specificities, accuracies, and diagnostic odds ratios were calculated. Subgroup analysis of diagnostic performance was conducted according to (1) gender (male versus female), (2) age (≤ median versus > median), (3) data source (local database versus public database), and (4) CT attenuation (lucent versus sclerotic or mixed). The alpha level for statistical tests was set as 0.05 and two-sided, unless stated otherwise.

## Results

### Patient and lesion characteristics

There were 223 positive cases and 105 negative cases, respectively (Table 1 and Supplementary Data S1). The difference in gender, and CT attenuation were not found among five databases (all *p* > 0.05), while age (*p* = 0.007), malignancy history (*p* = 0.021), presence of pain (*p* = 0.009), anatomical site (*p* < 0.001), and presence of fat (*p* = 0.001) showed significant difference among five databases.

**Table 1:** Patient and lesion characteristics

	Center A	Center B	Center C	Eurorad	Radiopeadia	Overall	*p*-value
No. of patients	39	68	71	36	114	328	
Age, mean ± standard deviation, median (range)	51.1 ± 18.1	39.1 ± 16.2	47.4 ± 14.9	46.8 ± 17.5	45.3 ± 19.1	45.3 ± 17.6	0.007
Gender, *n* (%)							0.147
Male	17 (43.6)	37 (54.4)	47 (66.2)	18 (50.0)	69 (60.5)	188 (57.3)	
Female	22 (564)	31 (45.6)	24 (33.8)	18 (50.0)	45 (39.5)	140 (42.7)	
Malignancy history, *n* (%)^a^							0.021
Present	4 (10.3)	1 (1.5)	12 (16.9)	4 (11.1)	11 (9.6)	32 (9.8)	
Absent	35 (89.7)	67 (98.5)	59 (83.1)	32 (88.9)	103 (90.4)	296 (90.2)	
Pain, *n* (%)							0.009
Present	28 (71.8)	42 (61.8)	34 (47.9)	29 (80.6)	67 (58.8)	200 (61.0)	
Absent	11 (28.2)	26 (38.2)	37 (52.1)	7 (19.4)	47 (41.2)	128 (39.0)	
Anatomical site, *n* (%)							< 0.001
Upper limb	7 (17.9)	20 (29.4)	2 (2.8)	5 (13.9)	17 (14.9)	51 (15.5)	
Lower limb	21 (53.8)	35 (51.5)	10 (14.1)	13 (36.1)	31 (27.2)	110 (33.5)	
Pelvic bone	6 (15.4)	9 (13.2)	10 (14.1)	10 (27.8)	15 (13.2)	50 (15.2)	
Spine	3 (7.7)	2 (2.9)	34 (47.9)	3 (8.3)	18 (15.8)	60 (18.3)	
Other	2 (5.1)	2 (2.9)	15 (21.1)	5 (13.9)	33 (28.9)	57 (17.4)	
Fat, *n* (%)							0.001
Present	39 (100.0)	68 (100.0)	71 (100.0)	31 (86.1)	112 (98.2)	321 (97.9)	
Absent	0 (0.0)	0 (0.0)	0 (0.0)	5 (13.9)	2 (1.8)	7 (2.1)	
Aggressive features, *n* (%)							0.001
Present	26 (66.7)	26 (38.2)	18 (25.4)	17 (47.2)	42 (36.8)	129 (39.3)	
Absent	13 (33.3)	42 (61.8)	53 (74.6)	19 (52.8)	72 (63.2)	199 (60.7)	
CT attenuation, *n* (%)							0.132
Lucent density	12 (30.8)	28 (41.2)	38 (53.5)	12 (33.3)	47 (41.2)	137 (41.8)	
Sclerotic or mixed density	27 (69.2)	40 (58.8)	33 (46.5)	24 (66.7)	67 (58.8)	191 (58.2)	

^a^ 32 patients with malignancy history included 10 patients with lung cancer, 4 patients with lymphoma, 4 patients with renal cell carcinoma, 2 patients with esophageal cancer, 2 patients with liver cancer, 1 patient with prostatic cancer 1 patient gastric cancer, 1 patient with thyroid cancer, and 7 patients with other malignancies. The imaging aggressive features include cortical involvement, soft tissue extension, pathologic fracture, or aggressive periosteal reaction

### Inter-reader agreement and diagnostic performance

**Fig. 2:**
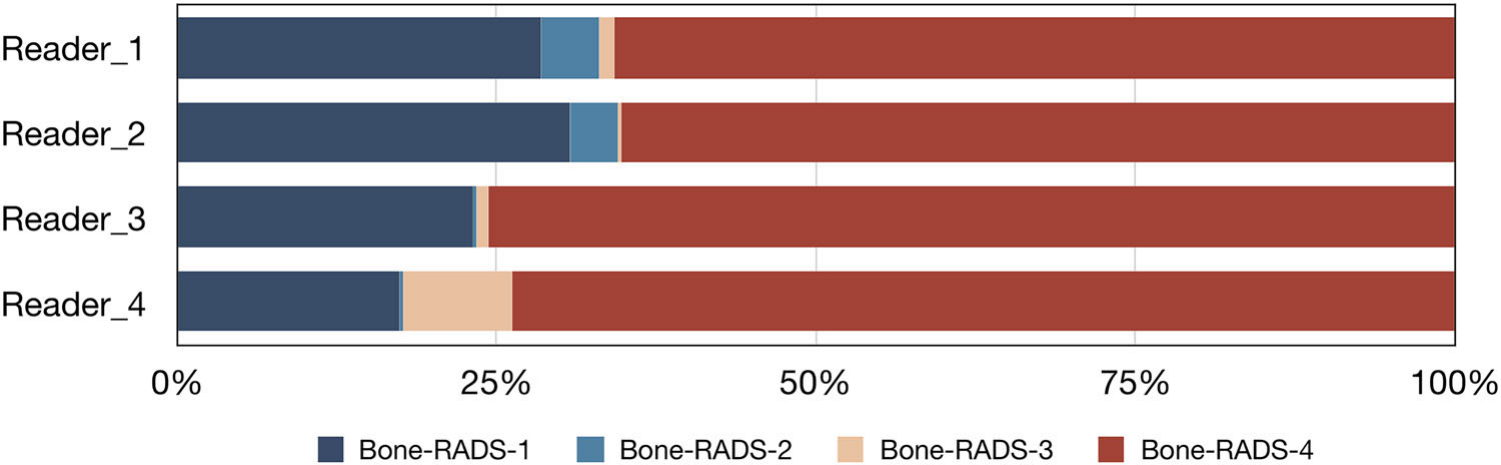
Bar plot of Bone-RADS rating according to readers

The 328 included lesions were categorized into Bone-RADS-1 to Bone-RADS-4, respectively (Table 2, Fig. 2, and Supplementary Data S2). Representative cases for Bone-RADS-1 and Bone-RADS-4 were provided (Figs. S1, S2). The inter-reader agreement between two musculoskeletal radiologists and two non-musculoskeletal radiologists was moderate (weighted kappa of 0.553 and 0.403). The diagnostic performance for identifying positive cases ranged according to radiologists with sensitivities of 88.8% to 94.6%, specificities of 42.9% to 71.1%, accuracies of 78.0% to 86.6%, and diagnostic odds ratios of 11.60 to 34.26 (Table 3). The diagnostic performance of Bone-RADS-CT did not seem to be significantly different between gender, age, source of data and CT attenuation. (Supplementary Table S1).

**Table 2:** Bone-RADS rating according to readers

	Negative	Positive	Overall
No. of cases	105	223	328
Reader 1			
Bone-RADS-1	69 (65.7)	25 (11.2)	94 (28.7)
Bone-RADS-2	4 (3.8)	11 (4.9)	15 (4.6)
Bone-RADS-3	0 (0.0)	2 (0.9)	2 (0.6)
Bone-RADS-4	32 (30.5)	185 (83.0)	217 (66.2)
Reader 2			
Bone-RADS-1	81 (77.1)	20 (9.0)	101 (30.8)
Bone-RADS-2	4 (3.8)	8 (3.6)	12 (3.7)
Bone-RADS-3	0 (0.0)	1 (0.4)	1 (0.3)
Bone-RADS-4	20 (19.0)	194 (87.0)	214 (65.2)
Reader 3			
Bone-RADS-1	56 (53.3)	20 (9.0)	76 (23.2)
Bone-RADS-2	1 (1.0)	0 (0.0)	1 (0.3)
Bone-RADS-3	1 (1.0)	2 (0.9)	3 (0.9)
Bone-RADS-4	47 (44.8)	201 (90.1)	248 (75.6)
Reader 4			
Bone-RADS-1	45 (42.9)	12 (5.4)	57 (17.4)
Bone-RADS-2	1 (1.0)	0 (0.0)	1 (0.3)
Bone-RADS-3	20 (19.0)	8 (8.5)	28 (8.5)
Bone-RADS-4	39 (37.1)	203 (73.8)	242 (73.8)

*Bone-RADS* Bone reporting and data system

**Table 3:** Diagnostic performance of Bone-RADS according to readers

	TP	FP	FN	TN	Sensitivity	Specificity	Accuracy	DOR
Reader 1	198	36	25	69	88.8%	65.7%	81.4%	15.18
Reader 2	203	24	20	81	91.0%	77.1%	86.6%	34.26
Reader 3	203	49	20	56	91.0%	53.3%	79.0%	11.60
Reader 4	211	60	12	45	94.6%	42.9%	78.0%	13.19

*TP* true positive (positive cases that diagnosed as Bone-RADS-2, 3, or 4), *FP* false positive (negative cases that diagnosed as Bone-RADS-2, 3, or 4) *FN* false negative (positive cases that diagnosed as Bone-RADS-1), *TN* true negative (negative cases that diagnosed as Bone-RADS-1), *DOR* diagnostic odds ratio, *Bone-RADS* Bone reporting and data system

## Discussion

**Fig. 3:**
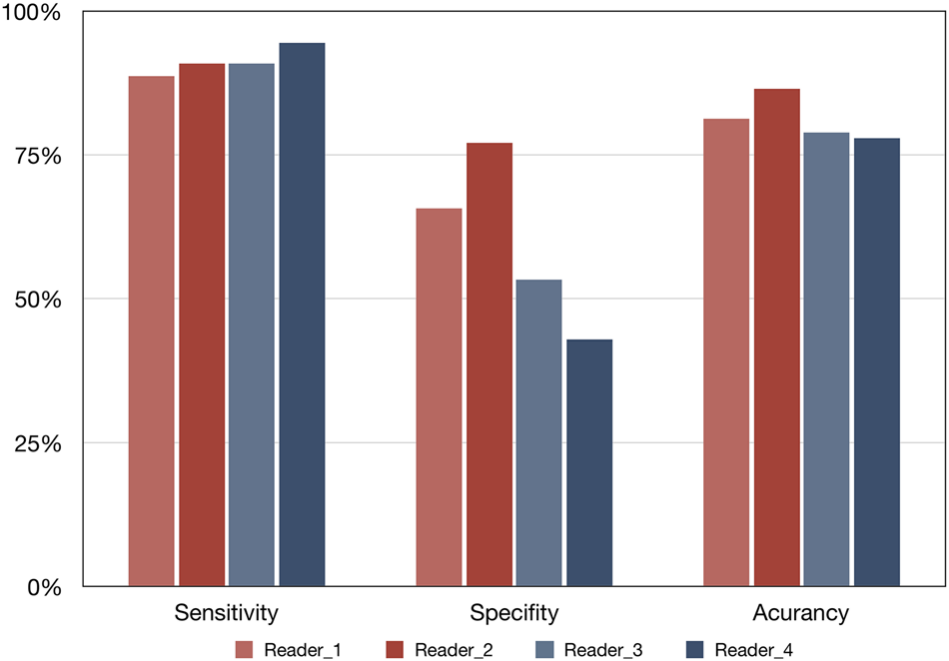
Histogram of diagnostic performance of Bone-RADS according to readers

Our study validated the Bone-RADS-CT for incidental solitary bone lesions in adults using three local and two public databases. Bone-RADS-CT demonstrated moderate inter-reader agreement between both musculoskeletal and non-musculoskeletal radiologists, and presented high sensitivity and diagnostic accuracy but relatively low specificity (Fig. 3).

The Bone-RADS-CT was validated by Park et al using a single-center dataset of 179 patients who underwent biopsy for bone lesions [25]. Two musculoskeletal radiologists with 5 and 6 years of experience demonstrated sensitivities of 94.7% and 82.4%, specificities of 10.8% and 10.8%, and accuracies of 57.3% and 50.5% for lucent lesions, respectively; and sensitivities of 75.0% and 67.9%, specificities of 27.1% and 27.1%, and accuracies of 44.7% and 42.1% for sclerotic or mixed lesions, respectively. Our study exhibited better diagnostic performance, with sensitivities of 91.0% to 97.3%, specificities of 46.2% to 92.3%, and accuracies of 84.7% to 92.7% for lucent lesions, and sensitivities of 86.6% to 92.0%, specificities of 41.8% to 72.2%, and accuracies of 71.2% to 82.2% for sclerotic or mixed lesions. We hypothesize that the sample may partially account for the difference. The biopsy cases may be challenging to diagnose based only on imaging appearance, whereas our study included more cases with typical presentation that are educationally significant and more cases with aggressive features (cortical involvement, soft tissue extension, pathologic fracture, or aggressive periosteal reaction) that are easier to diagnose based solely on imaging examinations. Another factor contributing to the difference could be the selection of representative images for evaluation. In our study, readers assessed the key image via a PowerPoint document to determine the Bone-RADS category, instead of reading the whole image set. This can potentially improve the diagnostic performance of the readers. A revised version of Bone-RADS excluded the original criteria for lesion-related pain and history of malignancy [27], resulting in improvement in specificity while maintaining the sensitivity. Some benign or intermediate lesions may present with pain but are classified as Bone-RADS-4 lesions by the original system, such as osteoid osteomas, chondroblastomas, and giant cell tumors of bone [2, 13]. The original system placed too much emphasis on the clinical appearance of pain and the history of malignancy, while the revised system has shifted the evaluation focus, enhancing the objective assessment of imaging findings over subjective symptoms, which can vary significantly at presentation.

The inter- and intra-reader reliability are also prerequisites for clinical use of Bone-RADS. Park et al demonstrated a good inter-reader reliability between two musculoskeletal radiologists with 5 and 6 years of experience (weighted kappa, 0.744) [25]. Ramadan et al showed even higher inter- and intra-reader reliability among three musculoskeletal radiologists with 9 to 12 years of experience (weighted kappa, 0.922 and 0.983) [26]. The revised Bone-RADS also improved the inter-reader reliability between two musculoskeletal and general radiologists with 10 and 5 years of experience, respectively (weighted kappa, 0.854) [27]. However, our inter-reader reliabilities were both moderate for musculoskeletal and non-musculoskeletal radiologists (weighted kappa, 0.553 and 0.403). Our readers were from a general hospital, but not a specialized division of musculoskeletal imaging and intervention. Consequently, our readers may have had insufficient exposure to cases to develop expertise in bone lesions. Radiologists with more experience in bone tumors may exhibit higher inter-reader reliability [25, 26]. Furthermore, our sample may include rarer diseases or common diseases with rare imaging appearances that are worth publishing as case reports. The opinion on these cases can be more diverse due to different levels of experience. Our future study aims to investigate the influence of training and experience on the inter-reader reliability of Bone-RADS. The potential improvement in inter-reader reliability by using the revised Bone-RADS also requires validation.

Bone-RADS offers several advantages. First, the Bone-RADS can be independently applied to either CT or MRI [24]. In contrast, OT-RADS relies on contrast-enhanced MRI [19, 22], and the BTI-RADS necessitates both CT and contrast-enhanced MRI for evaluation [23]. Bone-RADS is more clinically practicable when only a single imaging modality is available, regardless of whether it is contrast-enhanced or not. Second, the Bone-RADS white paper presents sample cases that demonstrate the pathways from imaging features to the actionable management recommendation [24]. These cases effectively illustrate the applicability of Bone-RADS algorithms on both CT and MRI. The ACR Bone-RADS for radiograph also provides sample cases for calculating the score and reaching a final grading [18]. These systems can be better understood and promoted with the aid of these documents. Third, the system is designed for a broader disease spectrum, including inflammation and infarction [24], whereas other systems restrict their application to bone tumors [17–19, 22, 23]. This is essential because incidental bone lesions are not always bone tumors [31], and infections can sometimes be a crucial differential diagnosis for malignant bone tumors [32].

Despite these advantages, Bone-RADS still has room for improvement. First, Bone-RADS algorithms heavily rely on specific imaging characteristics of bone tumors [24]. To use Bone-RADS, the readers should have enough experience to tell whether a lesion is entirely or clearly consistent with more than twenty specific bone tumors. This would be challenging for readers with insufficient expertise, as the system is expected to be used more widely outside musculoskeletal practice. OT-RADS has a similar issue, where the readers need to remember typical MRI characteristics of a series of bone tumors and their OT-RADS classification to make a recommendation [21, 22]. On the other hand, it is more user-friendly for readers to determine the rating of a lesion according to scores of imaging features [17, 18, 23]. The readers only need basic knowledge of the assessment of imaging features, rather than the knowledge needed to reach a histological diagnosis. Second, Bone-RADS combines the malignancy potential and recommendation into the grading system [24]. While this is expected to be more clinically practical, it may confuse users. Other systems follow the common convention that a larger number indicates a higher malignancy potential or severity [17, 18, 21–23, 26, 33]. However, the Bone-RADS-2 means incomplete imaging in the Bone-RADS system, but a higher malignancy possibility or severity than Bone-RADS-1. It should be set as Bone-RADS-0, similar to other systems for bone tumors [18, 21, 22]. Third, the classification for bone lesions is not entirely appropriate. It may not always be suitable to treat infarction as Bone-RADS-1 lesions and leave alone, as they are sometimes curable and require referral for management [34, 35]. Further, not only intermediate lesion should be managed with follow-up. Fibrous dysplasia and enchondroma, although classified as Bone-RADS-1 lesions, may transform into malignancies and sometimes also need follow-ups [36, 37]. We believe that these points need to be considered when updating Bone-RADS. In addition to these drawbacks, Bone-RADS remains a timely tool for bone lesions in the era of structured reporting [38, 39]. We believe that Bone-RADS will someday become a robust system with sufficient scientific evidence through a step-by-step, structured and systematic approach [40].

Our study has the following limitations. First, the Bone-RADS is designed for incidental solitary bone lesions in adults that are detected in imaging examinations for an unrelated reason. However, it is hard to determine whether a lesion is incidental in a retrospective study design [25–27]. Therefore, we included all solitary bone lesions, regardless of their nature. A prospective study is warranted to test the diagnostic performance of Bone-RADS under an incidental condition. Second, our sample does not present the epidemiology of the bone lesions. The benign lesions may be underreported due to their limited clinical significance and may therefore not be identified during the sample screening. The rare histological types of bone lesions and common ones with rare imaging appearances are more likely to be recorded for educational and research purposes and published as case reports. Third, our study only allowed the radiologists to evaluate the lesions with several representative images, rather than continuous image series via the picture archiving and communication system. However, we believe the current settings are efficient enough for clinical validation for Bone-RADS and can provide insights for improvements [29]. We included CT images with a slice thickness of at least 5 mm. Further investigation is warranted to determine whether the slice thickness affects the diagnostic performance of Bone-RADS-CT. Finally, our study only assessed the diagnostic performance and inter-reader reliability of Bone-RADS. The potential influence of Bone-RADS on the clinical procedure and subsequent management remains unclear. Future studies may implement Bone-RADS in a prospective manner and investigate whether it can influence management and outcomes, as well as cost-effectiveness.

In conclusion, our study found that Bone-RADS-CT were of moderate inter-reader reproducibility and high sensitivity and accuracy but relatively low specificity. We encourage future prospective studies to validate its usefulness, and to provide insights for improvements. Nevertheless, it is a great and timely attempt for a systematic and standardized approach evaluation of bone lesions.

## Supplementary information

ELECTRONIC SUPPLEMENTARY MATERIAL

## Data Availability

All data generated or analyzed during this study are included in this published article and its additional files.

## Abbreviations

Bone-RADS: Bone reporting and data system
BTI-RADS: Bone tumor imaging reporting and data system
OT-RADS: Osseous tumor reporting and data system
REST: Radiological evaluation score for bone tumors

## References

[1] Blackburn CW, Richardson SM, DeVita RR et al (2023) What is the prevalence of clinically important findings among incidentally found osseous lesions? Clin Orthop Relat Res 481:1993–2002. 10.1097/CORR.0000000000002630 36975798 10.1097/CORR.0000000000002630 PMC10499109

[2] Costelloe CM, Madewell JE (2013) Radiography in the initial diagnosis of primary bone tumors. AJR Am J Roentgenol 200:3–7. 10.2214/AJR.12.8488 23255735 10.2214/AJR.12.8488

[3] Ladd LM, Roth TD (2017) Computed tomography and magnetic resonance imaging of bone tumors. Semin Roentgenol 52:209–226. 10.1053/j.ro.2017.04.006 28965542 10.1053/j.ro.2017.04.006

[4] Hoffman RJ, Stanborough RO, Garner HW (2022) Diagnostic imaging approach to solitary bone lesions. Semin Roentgenol 57:241–251. 10.1053/j.ro.2022.01.005 35842245 10.1053/j.ro.2022.01.005

[5] Gondim Teixeira PA, Lemore A, Vogt N et al (2023) Initial evaluation of focal bone lesions: how do we do it? Semin Musculoskelet Radiol 27:471–479. 10.1055/s-0043-1769775 37748471 10.1055/s-0043-1769775

[6] Gondim Teixeira PA, Lombard C, Moustache-Espinola P et al (2023) Initial characterization of focal bone lesions with conventional radiographs or computed tomography: diagnostic performance and interobserver agreement assessment. Can Assoc Radiol J 74:404–414. 10.1177/08465371221131755 36207066 10.1177/08465371221131755

[7] WHO Classification of Tumours Edition Board (2020) World Health Organization classification of tumours: WHO classification of tumours of soft tissue and bone, 5th edn. IARC Press, Lyon

[8] Sung H, Ferlay J, Siegel RL et al (2021) Global cancer statistics 2020: GLOBOCAN estimates of incidence and mortality worldwide for 36 cancers in 185 countries. CA Cancer J Clin 71:209–249. 10.3322/caac.21660 33538338 10.3322/caac.21660

[9] Siegel RL, Giaquinto AN, Jemal A (2024) Cancer statistics, 2024. CA Cancer J Clin 74:12–49. 10.3322/caac.21820 38230766 10.3322/caac.21820

[10] Strauss SJ, Frezza AM, Abecassis N et al (2021) Bone sarcomas: ESMO-EURACAN-GENTURIS-ERN PaedCan clinical practice guideline for diagnosis, treatment and follow-up. Ann Oncol 32:1520–1536. 10.1016/j.annonc.2021.08.1995 34500044 10.1016/j.annonc.2021.08.1995

[11] National Comprehensive Cancer Network (2024) NCCN clinical practice guidelines in oncology: bone cancer, version 1.2025. Available via https://www.nccn.org/professionals/physician_gls/pdf/bone.pdf. Accessed 05 Sep 2024

[12] Siegel GW, Biermann JS, Chugh R et al (2015) The multidisciplinary management of bone and soft tissue sarcoma: an essential organizational framework. J Multidiscip Healthc 8:109–115. 10.2147/JMDH.S49805 25733913 10.2147/JMDH.S49805 PMC4340372

[13] Mehta K, McBee MP, Mihal DC, England EB (2017) Radiographic analysis of bone tumors: a systematic approach. Semin Roentgenol 52:194–208. 10.1053/j.ro.2017.04.002 28965541 10.1053/j.ro.2017.04.002

[14] Burnside ES, Sickles EA, Bassett LW et al (2009) The ACR BI-RADS experience: learning from history. J Am Coll Radiol 6:851–860. 10.1016/j.jacr.2009.07.023 19945040 10.1016/j.jacr.2009.07.023 PMC3099247

[15] An JY, Unsdorfer KML, Weinreb JC (2019) BI-RADS, C-RADS, CAD-RADS, LI-RADS, Lung-RADS, NI-RADS, O-RADS, PI-RADS, TI-RADS: reporting and data systems. Radiographics 39:1435–1436. 10.1148/rg.2019190087 31498744 10.1148/rg.2019190087 PMC7251936

[16] Ribeiro GJ, Gillet R, Blum A, Teixeira PAG (2023) Imaging report and data system (RADS) for bone tumors: where do we stand and future directions. Skelet Radiol 52:151–156. 10.1007/s00256-022-04179-2 10.1007/s00256-022-04179-2 36074158

[17] Salunke AA, Nandy K, Puj K et al (2022) A proposed “Radiological Evaluation Score for Bone Tumors” (REST): an objective system for assessment of a radiograph in patients with suspected bone tumor. Musculoskelet Surg 106:371–382. 10.1007/s12306-021-00711-0 34125399 10.1007/s12306-021-00716-9

[18] Caracciolo JT, Ali S, Chang CY et al (2023) Bone tumor risk stratification and management system: a consensus guideline from the ACR bone reporting and data system committee. J Am Coll Radiol 20:1044–1058. 10.1016/j.jacr.2023.07.017 37855758 10.1016/j.jacr.2023.07.017

[19] Kim Y, Chee CG, Kang Y (2025) Validation of the American College of Radiology Bone Reporting and Data System™ (ACR Bone-RADS™) for classifying osteolytic bone tumors. Skelet Radiol 54:1841–1850. 10.1007/s00256-025-04881-x 10.1007/s00256-025-04881-x 39894855

[20] Park SY, Yoon MA, Lee MH et al (2025) Validation of American College of Radiology Bone Reporting and Data System (Bone-RADS) version 2023 for diagnosis of malignant tumors of appendicular bone on conventional radiographs. Eur J Radiol 183:111861. 10.1016/j.ejrad.2024.111861 39637582 10.1016/j.ejrad.2024.111861

[21] Chhabra A, Gupta A, Thakur U et al (2021) Osseous tumor reporting and data system—multireader validation study. J Comput Assist Tomogr 45:571–585. 10.1097/RCT.0000000000001184 34270485 10.1097/RCT.0000000000001184

[22] Guirguis M, Gupta A, Thakur U et al (2023) Osseous-tissue tumor reporting and data system with diffusion-weighted imaging of bone tumors—an interreader analysis and whether it adds incremental value on tumor grading over conventional magnetic resonance imaging. J Comput Assist Tomogr 47:255–263. 10.1097/RCT.0000000000001415 36877760 10.1097/RCT.0000000000001415

[23] Ribeiro GJ, Gillet R, Hossu G et al (2021) Solitary bone tumor imaging reporting and data system (BTI-RADS): initial assessment of a systematic imaging evaluation and comprehensive reporting method. Eur Radiol 31:7637–7652. 10.1007/s00330-021-07745-9 33765161 10.1007/s00330-021-07745-9

[24] Chang CY, Garner HW, Ahlawat S et al (2022) Society of Skeletal Radiology—white paper. Guidelines for the diagnostic management of incidental solitary bone lesions on CT and MRI in adults: bone reporting and data system (Bone-RADS). Skelet Radiol 51:1743–1764. 10.1007/s00256-022-04022-8 10.1007/s00256-022-04022-8 PMC9283187 35344076

[25] Park C, Azhideh A, Pooyan A et al (2024) Diagnostic performance and inter-reader reliability of bone reporting and data system (Bone-RADS) on computed tomography. Skelet Radiol 54:209–217. 10.1007/s00256-024-04721-4 10.1007/s00256-024-04721-4 38853160

[26] Ramadan ZA, Elmorsy AH, Taman SE et al (2024) Inter-observer and intra-observer agreement of bone reporting and data system (Bone-RADS) in the interpretation of bone tumors on computed tomography. Clin Imaging 117:110367. 10.1016/j.clinimag.2024.110367 39602845 10.1016/j.clinimag.2024.110367

[27] Haseli S, Park C, Azhideh A et al (2025) Performance and reliability comparison: original vs. revised bone reporting and data system (Bone-RADS). Skelet Radiol. 10.1007/s00256-025-04865-x 10.1007/s00256-025-04865-x 39838067

[28] Monti CB, Ambrogi F, Sardanelli F (2024) Sample size calculation for data reliability and diagnostic performance: a go-to review. Eur Radiol Exp 8:79. 10.1186/s41747-024-00474-w 38965128 10.1186/s41747-024-00474-w PMC11224179

[29] Chhabra A, Alaia EF, Ashikyan O et al (2024) MSKI-RADS: an MRI-based musculoskeletal infection reporting and data system for the diagnosis of extremity infections. Radiology 312:e232914. 10.1148/radiol.232914 39189902 10.1148/radiol.232914

[30] Gisev N, Bell JS, Chen TF (2013) Interrater agreement and interrater reliability: key concepts, approaches, and applications. Res Social Adm Pharm 9:330–338. 10.1016/j.sapharm.2012.04.004 22695215 10.1016/j.sapharm.2012.04.004

[31] Zhong J (2024) Deep learning-based diagnostic models for bone lesions: is current research ready for clinical translation? Eur Radiol 34:4284–4286. 10.1007/s00330-023-10555-w 38189983 10.1007/s00330-023-10555-w PMC11213795

[32] Henninger B, Glodny B, Rudisch A et al (2013) Ewing sarcoma versus osteomyelitis: differential diagnosis with magnetic resonance imaging. Skelet Radiol 42:1097–1104. 10.1007/s00256-013-1632-5 10.1007/s00256-013-1632-5 23685708

[33] Messiou C, Hillengass J, Delorme S et al (2019) Guidelines for acquisition, interpretation, and reporting of whole-body MRI in myeloma: myeloma response assessment and diagnosis system (MY-RADS). Radiology 291:5–13. 10.1148/radiol.2019181949 30806604 10.1148/radiol.2019181949

[34] Talusan PG, Diaz-Collado PJ, Reach JS Jr (2014) Freiberg’s infraction: diagnosis and treatment. Foot Ankle Spec 7:52–56. 10.1177/1938640013510314 24319044 10.1177/1938640013510314

[35] Sodhi N, Acuna A, Etcheson J et al (2020) Management of osteonecrosis of the femoral head. Bone Joint J 102-B:122–128. 10.1302/0301-620X.102B7.BJJ-2019-1611.R1 32600203 10.1302/0301-620X.102B7.BJJ-2019-1611.R1

[36] Qu N, Yao W, Cui X, Zhang H (2015) Malignant transformation in monostotic fibrous dysplasia: clinical features, imaging features, outcomes in 10 patients, and review. Medicine (Baltimore) 94:e369. 10.1097/MD.0000000000000369 25621678 10.1097/MD.0000000000000369 PMC4602648

[37] Herget GW, Strohm P, Rottenburger C et al (2014) Insights into enchondroma, enchondromatosis and the risk of secondary chondrosarcoma: review of the literature with an emphasis on the clinical behaviour, radiology, malignant transformation and the follow up. Neoplasma 61:365–378. 10.4149/neo_2014_046 24645839 10.4149/neo_2014_046

[38] Chhabra A (2023) Letter to editor in reference to OT-RADS. Skelet Radiol 52:771–772. 10.1007/s00256-022-04209-z 10.1007/s00256-022-04209-z 36260153

[39] Ribeiro GJ, Teixeira PAG (2023) Response to the letter to the editor in reference to OT-RADS. Skelet Radiol 52:769. 10.1007/s00256-022-04210-6 10.1007/s00256-022-04210-6 36260152

[40] Dabi Y, Rockall A, Sadowski E, et al (2024) O-RADS MRI to classify adnexal tumors: from clinical problem to daily use. Insights Imaging 15:29. 10.1186/s13244-023-01598-0 38289563 10.1186/s13244-023-01598-0 PMC10828223

