# Supplementary material for: Bone reporting and data system on CT (Bone-RADS-CT): a validation study by four readers on 328 cases from three local and two public databases
Source: Insights Imaging. 2025 Aug 12;16:174. doi: 10.1186/s13244-025-02057-8 (PMC12344047; doi:10.1186/s13244-025-02057-8)
Supplement: Supplementary file 1 — ELECTRONIC SUPPLEMENTARY MATERIAL [file 13244_2025_2057_MOESM1_ESM.pdf]

Bone reporting and data system on CT (Bone-RADS-CT): a validation study by four readers on 328 cases from three local and two public databases

ELECTRONIC SUPPLEMENTARY MATERIAL

Supplementary Note S1 Sample selection process
Supplementary Note S2 Sample size calculation
Supplementary Note S3 Methodology for establishment of final diagnosis and reference standard of Bone-RADS
Supplementary Note S4 Methodology for training and formal evaluation

Supplementary Table S1 Subgroup analysis of diagnostic performance

Supplementary Figure S1 Representative Bone-RADS-1 cases
Supplementary Figure S2 Representative Bone-RADS-4 cases

Supplementary Note S1 Sample selection process

1. Center A database

Data source: Center A (Tongren Hospital, Shanghai Jiao Tong University School of Medicine) is a local tertiary hospital affiliated of a university with more than 1200 beds. The volume of outpatient per year is 2 million, and the volume of inpatient per year is 60 thousand. The hospital is a general comprehensive hospital without a musculoskeletal cancer center. The study has been approved by the institutional review board, and the written informed consent from participants was waived.

Data period: 01 Jan 2019 and 30 Jun 2023

Data selection: The picture archiving and communication system was searched by using terms related to bone lesions according to the method proposed by Chhabra et al (https://doi.org/10.1097/RCT.0000000000001184) and Guirguis et al (https://doi.org/10.1097/RCT.0000000000001415). The eligibility of 865 potential cases was evaluated by our group. The cases should fulfill the following criteria: (a) >=18 years old; (b) solitary bone lesions; (c) complete CT examination with slice thickness of at least 5 mm, and appropriate window width and level for bone and soft tissue evaluation; (d) clinical history available; and (e) histologically or clinically confirmed diagnosis, or with typical appearance of "do not touch" lesions and remain stable for at least two years. The following cases would be excluded: (a) overlapping cases; (b) follow-up examinations; (c) examinations after treatment; (d) unsatisfied image quality; (e) not allowed for non-commercial use.

Data inclusion: In 865 potential cases, the following cases did not meet the inclusion criteria: (a) >=18 years old (n = 61); (b) solitary bone lesions (n = 235) (c) complete CT examination with slice thickness of at least 5 mm, and appropriate window width and level for bone and soft tissue evaluation (n = 27); (d) clinical history available (n = 266). The following cases were excluded during further assessment: (a) overlapping cases (n = 3); (b) follow-up examinations (n = 82); (c) examinations after treatment (n = 129); (d) unsatisfied image quality (n = 23); (e) not allowed for non-commercial use (n = 0). Finally, 39 cases were included.

2. Center B database

Data source: Center B (Shanghai Sixth People's Hospital, Shanghai Jiao Tong University School of Medicine) is a local tertiary hospital affiliated of a university with more than 2400 beds. The volume of outpatient per year is 5 million, and the volume of inpatient per year is 150 thousand. The hospital is a general comprehensive hospital with a musculoskeletal cancer center. The study has been approved by the institutional review board, and the written informed consent from participants was waived.

Data period: 01 Jan 2018 and 30 Jun 2020

Data selection: The 183 potential cases confirmed by histology were collected for educational purpose by an orthopedist (GCZ) and a pathologist (QY). The orthopedist and the pathologist collected these cases for lectures on bone lesions. These cases were assessed by our group. The cases should fulfill the following criteria: (a) >=18 years old; (b) solitary bone lesions; (c) complete CT examination with slice thickness of at least 5 mm, and appropriate window width and level for bone and soft tissue evaluation; (d) clinical history available; and (e) histologically or clinically confirmed diagnosis, or with typical appearance of "do not touch" lesions and remain stable for at least two years. The following cases would be excluded: (a) overlapping cases; (b) follow-up examinations; (c) examinations after treatment; (d) unsatisfied image quality; (e) not allowed for non-commercial use.

Data inclusion: In 183 potential cases, the following cases did not meet the inclusion criteria: (a) >=18 years old (n = 14); (b) solitary bone lesions (n = 19); (c) complete CT examination with slice thickness of at least 5 mm, and appropriate window width and level for bone and soft tissue evaluation (n = 46); (d) clinical history available (n = 24). The following cases were excluded during further assessment: (a) overlapping cases (n = 0); (b) follow-up examinations (n = 2); (c) examinations after treatment (n = 7); (d) unsatisfied image quality (n = 3); (e) not allowed for non-commercial use (n = 0). Finally, 68 cases were included.

3. Center C database

Data source: Center C (Universal Medical Imaging Diagnostic Center, Shanghai) is a local medical imaging center providing medical imaging service including CT scan, MRI scan, PET-CT scan, PET-MRI scan, etc. The study has been approved by the institutional review board, and the written informed consent from participants was waived.

Data period: 01 Jan 2010 and 31 Dec 2015

Data selection: The 181 potential cases confirmed by histology were collected by a radiologist (CZL) for research purpose. The radiologist collected these cases to investigate the presentation of the bone and soft tissue lesions on CT, MRI, and PET. These cases were assessed by our group. The cases should fulfill the following criteria: (a) >=18 years old; (b) solitary bone lesions; (c) complete CT examination with slice thickness of at least 5 mm, and appropriate window width and level for bone and soft tissue evaluation; (d) clinical history available; and (e) histologically or clinically confirmed diagnosis, or with typical appearance of "do not touch" lesions and remain stable for at least two years. The following cases would be excluded: (a) overlapping cases; (b) follow-up examinations; (c) examinations after treatment; (d) unsatisfied image quality; (e) not allowed for non-commercial use.

Data inclusion: In 181 potential cases, the following cases did not meet the inclusion criteria: (a) >=18 years old (n = 17); (b) solitary bone lesions (n = 27); (c) complete CT examination with slice thickness of at least 5 mm, and appropriate window width and level for bone and soft tissue evaluation (n = 0); (d) clinical history available (n = 16). The following

cases were excluded during further assessment: (a) overlapping cases (n = 0); (b) follow-up examinations (n = 35); (c) examinations after treatment (n = 12); (d) unsatisfied image quality (n = 3); (e) not allowed for non-commercial use (n = 0). Finally, 71 cases were included.

4. Eurorad database
Data source: Eurorad (https://www.eurorad.org) is a free, public, peer-reviewed radiological case database established by European Society of Radiology. The cases from Eurorad are under the Creative Commons License CC BY-NC-SA 4.0 after July 2015. We have discussed the use of the cases in our study with the Eurorad team, and acquired the permission for use. We used them according to the "Terms and conditions for the use of Eurorad" (https://www.eurorad.org/terms-and-conditions).
Data period: the establish of the database to 30 Jun 2024
Data selection: The 384 potential cases were identified by using the advanced search of musculoskeletal system and imaging modality of CT. These filters allowed us to quickly identify potential cases in the musculoskeletal system with CT available. These cases were assessed by our group. The cases should fulfill the following criteria: (a) >=18 years old; (b) solitary bone lesions; (c) complete CT examination with slice thickness of at least 5 mm, and appropriate window width and level for bone and soft tissue evaluation; (d) clinical history available; and (e) histologically or clinically confirmed diagnosis, or with typical appearance of "do not touch" lesions and remain stable for at least two years. The following cases would be excluded: (a) overlapping cases; (b) follow-up examinations; (c) examinations after treatment; (d) unsatisfied image quality; (e) not allowed for non-commercial use.
Data inclusion: In 384 potential cases, the following cases did not meet the inclusion criteria: (a) >=18 years old (n = 31); (b) solitary bone lesions; (n = 201, which are mainly soft tissue lesions, fracture, and multiple lesions); (c) complete CT examination with slice thickness of at least 5 mm, and appropriate window width and level for bone and soft tissue evaluation (n = 39); (d) clinical history available (n = 19). The following cases were excluded during further assessment: (a) overlapping cases (n = 0); (b) follow-up examinations (n = 4); (c) examinations after treatment (n = 18); (d) unsatisfied image quality (n = 0); (e) not allowed for non-commercial use (n = 36, which are before July 2015). Finally, 36 cases were included.

5. Radiopeadia database
Data source: Radiopaedia (https://radiopaedia.org) is a rapidly growing, peer-reviewed open-edit radiology resource, compiled by radiologists and other health professionals from across the globe, involving a free, public, peer-reviewed radiological case database. The cases from Radiopaedia are under the Creative Commons License CC BY-NC-SA 3.0. We have discussed the use of the cases of our study with the Radiopaedia team, and acquired the permission for use. We used them according to the "Terms of use" (https://radiopaedia.org/terms) and licensing (https://radiopaedia.org/licence) of Radiopaedia.
Data period: the establish of the database to 30 Jun 2024
Data selection: The 2741 potential cases were distinguished by using the filter of musculoskeletal system, imaging modality of CT, and certain diagnosis. These filters allowed us to quickly identify potential cases in the musculoskeletal system with CT available. We further applied a filter of certain diagnosis to identify the cases with certain diagnosis but not those with probably and uncertain diagnosis. These cases were assessed by our group. The cases should fulfill the following criteria: (a) >=18 years old; (b) solitary bone lesions; (c) complete CT examination with slice thickness of at least 5 mm, and appropriate window width and level for bone and soft tissue evaluation; (d) clinical history available; and (e) histologically or clinically confirmed diagnosis, or with typical appearance of "do not touch" lesions and remain stable for at least two years. The following cases would be excluded: (a) overlapping cases; (b) follow-up examinations; (c) examinations after treatment; (d) unsatisfied image quality; (e) not allowed for non-commercial use.
Data inclusion: In 2029 potential cases, the following cases did not meet the inclusion criteria: (a) >=18 years old (n = 79); (b) solitary bone lesions (n = 1756, which are mainly soft tissue lesions, fracture, and multiple lesions); (c) complete CT examination with slice thickness of at least 5 mm, and appropriate window width and level for bone and soft tissue evaluation (n = 6); (d) clinical history available (n = 21). The following cases were excluded during further assessment: (a) overlapping cases (n = 0); (b) follow-up examinations (n = 19); (c) examinations after treatment (n = 32); (d) unsatisfied image quality (n = 2); (e) not allowed for non-commercial use (n = 0). Finally, 114 cases were included.

Supplementary Note S2 Sample size calculation

We did not perform *a priori* sample size calculation, but collected all the available cases from the local institutions and public databases. We performed a *post hoc* sample size calculation to confirm the appropriateness of our study (https://wnarifin.github.io/ssc/sssnsp.html). We used the overall sensitivity of 91.4%, specificity of 59.7%, overall malignancy rate of 68.0% for the calculation. The precision (10.0%), confidence level (95%), and drop rate (0%) were set. The calculation resulted 45 cases for sensitivity, and 289 cases for specificity, while the sample size of our study is 328 cases.

Of note, in observational studies sample size is often determined by feasibility or economic reasons (Eur Radiol Experimental, 2024, 8:79, https://doi.org/10.1186/s41747-024-00474-w). In this case it is possible to use the online calculator to calculate what will be the margin of error (precision) in correspondence of a specific sample size (https://wnarifin.github.io/ssc/sssnsp.html). In our study with a sample size of 328 cases, the precision for sensitivity is about 0.037, and the precision for specificity is about 0.095, both less than a widely-accepted cutoff of 0.10.

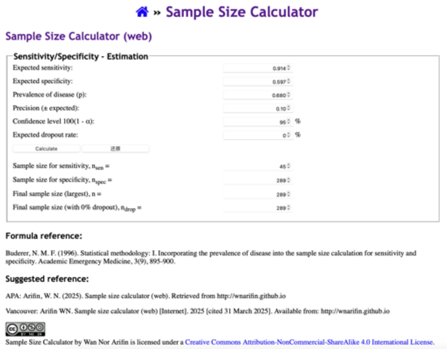

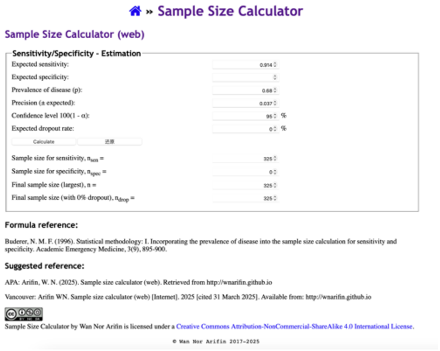

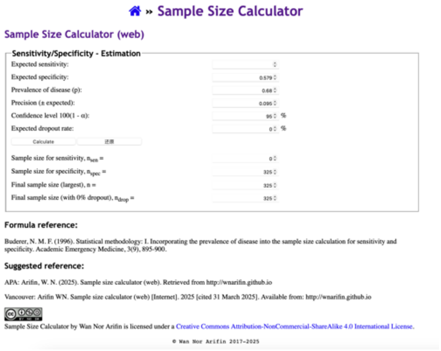

Supplementary Note S3 Methodology for establishment of final diagnosis and reference standard of Bone-RADS

1. Study group

Our study group was a multidisciplinary group that was composed of six radiologists with 6 to 32 years of experience (JYZ, YX, LJL, CZL, HZ, and WWY), one orthopedist with 6 years of experience (GCZ), one oncologist with 6 years of experience (SQM), and one pathologist with 6 years of experience (QY). Four out of six radiologists (JYZ, YX, LJL, and WWY) are musculoskeletal radiologists, one is an abdominal radiologist (HZ), and one is a radiologist in nuclear medicine (CZL). Four extra radiologists (YFH, DFD, XWL, and SD) were served as the readers for testing the Bone-RADS. Other members of our study groups included one biostatistical expert (LJJ), one biomedical engineering expert (JRY), and one MR research collaboration scientist (YS), who provided methodological suggestions.

2. Final diagnosis

The reference standard for the Bone-RADS-1 and Bone-RADS-4 lesions was established according to the final diagnosis of the case. The final diagnosis was reached by the multidisciplinary group according to the histological or clinically confirmation, typical appearance of “do not touch” lesions, and follow-ups for at least two years. We only established the reference standard for Bone-RADS-1 and Bone-RADS-4 lesions, since one of the major goals of the panel when creating these algorithms was to be certain that lesions designated Bone-RADS1 are truly benign processes that require no additional workup. Also, the panel feels that dismissing a malignant lesion has greater consequences than over-imaging a benign lesion; therefore, we have erred on the side of not ignoring Bone-RADS4 lesions.

For the Center A and Center C database, the final diagnosis was established by histological or clinically confirmation, typical appearance of “do not touch” lesions, and follow-ups for at least two years. For the Center B database, all the cases were confirmed by histology, and therefore the final diagnosis was reached according to the histology. Then, the standard reference was established according to the final diagnosis. For the Eurorad and Radiopeadia databases, the Bone-RADS-1 and Bone-RADS-4 ratings were established according to the final diagnosis in Eurorad, and the “certain” diagnosis in Radiopeadia.

2. Reference standard for Bone-RADS

In the Bone-RADS, some of the tumors have a definite Bone-RADS rating, and they were rated accordingly to established the reference standard. The tumors were rated as Bone-RADS-1, including (lucent lesion: fibrous dysplasia, non-ossifying fibroma, enchondroma, subchondral cyst, hemangioma, intraosseous lipoma; sclerotic or mixed lesion: non-ossifying fibroma, enostosis, sclerosing bone dysplasia, hemangioma, intraosseous lipoma, fibrous dysplasia, enchondroma < 5cm). The tumors were rated as Bone-RADS-4 (lucent lesion: osteoid osteoma, osteoblastoma, cortical metastasis, aneurysmal bone cyst, simple bone cyst, giant cell tumor of bone, chondroblastoma, clear cell chondrosarcoma, myeloma, eosinophilic granuloma, inflection; sclerotic or mixed lesion: enchondroma > 5cm, metastasis, chondrosarcoma, clear cell chondrosarcoma, myeloma, eosinophilic granuloma, inflection, atypical cartilaginous lesion).

Other tumors or disease did not present in the Bone-RADS were rated as follows. The reference standard of these tumors or disease for Bone-RADS was established by (1) Bone-RADS-1 or Bone-RADS-4 for histological benign, or malignant and intermediate confirmation, if it is available; (2) Bone-RADS-4 for clinical confirmation for those needs treatment, e. g. infections; (3) Bone-RADS-1 for benign lesion with follow-up for at least two years, and Bone-RADS-4 for malignancy lesions with progression during follow-up; (4) Bone-RADS-1 for benign lesion with typical appearance of “do not touch” lesions evaluated by two musculoskeletal radiologists with experience up to 32 years.

We did not establish the reference standard for the Bone-RADS-2 or Bone-RADS-3 lesions, since its suitability may change according to the experience and opinion of readers as well as attitude and socioeconomic status of the patients. An extra imaging procedure may be necessary for an inexperienced radiologist to reach the correct diagnosis, while follow-up may be preferred by a patient with fibrous dysplasia due to the possibility of malignant transformation. For the evaluation of diagnostic performance, we defined the cases as follows: TP = true positive, Bone-RADS-4 cases that diagnosed as Bone-RADS-2, 3, or 4; FP = false positive, Bone-RADS-1 cases that diagnosed as Bone-RADS-2, 3, or 4; FN = false negative, Bone-RADS-4 cases that diagnosed as Bone-RADS-1; TN = true negative, Bone-RADS-1 cases that diagnosed as Bone-RADS-1. These evaluations allow us to tell whether the Bone-RADS is effective for certainly confirm that lesions designated Bone-RADS1 are truly benign processes that require no additional work-up; meanwhile effective for not ignoring Bone-RADS4 lesions that are spacious for malignancy or need for treatment.

Supplementary Note S4 Methodology for training and formal evaluation

1. Study group

The study group is consisted of a group of readers who did not involve in the sample selection and reference standard establishment, and a group of experts with different clinical backgrounds who selected the sample for evaluation and established the reference standard for diagnostic performance analysis. For the image evaluation, there were four readers, including two musculoskeletal radiologists with 6 and 8 years of experience (YFH and DFD), and two non-musculoskeletal radiologists both with 9 years of experience (SD and XWL), respectively. For sample selection and reference standard establishment, there was a multidisciplinary group that was composed of six radiologists with 6 to 32 years of experience (JYZ, YX, LJL, CZL, HZ, and WWY), one orthopedist with 6 years of experience (GCZ), one oncologist with 6 years of experience (SQM), and one pathologist with 6 years of experience (QY). Four out of six radiologists (JYZ, YX, LJL, and WWY) are musculoskeletal radiologists, one is an abdominal radiologist (HZ), and one is a radiologist in nuclear medicine (CZL). Other members of our study groups included one biostatistical expert (LJJ), one biomedical engineering expert (JRY), and one MR research collaboration scientist (YS), who provided methodological suggestions.

2. Training phase of Bone-RADS

Before the formal assessment, the four readers studied the Bone-RADS algorithm document by themselves, via presentations prepared by the multidisciplinary group, and discussions with each other. Then, they were tested for their comprehension of the document by using ten attached representative cases to confirm that all the readers have a shared understanding of Bone-RADS. The uncertainties were solved by discussion with a multidisciplinary group. As the Bone-RADS on CT and MRI are introduced in the same white paper proposed by the Society of Skeletal Radiology, we studies the Bone-RADS on CT and MRI together.

The readers firstly studied the scope of development of Bone-RADS, the definition of "incidental solitary bone lesions in adults", the definition of lucent, sclerotic and mixed density lesions on CT, the definition of T1 hyperintense, T1 isointense/hypointense, T2 hypointense and T2 hyperintense lesions on MRI. They were told that the Bone-RADS is a bone reporting and data system with four possible diagnostic management recommendations. The example figures from the white paper were presented to the readers to allow a direct feeling of these concepts. The key concepts and frequently asked questions for Bone-RADS are summarized as follows. Although the current study did not involve the lesions on MRI, the related definitions were provided to completely present the Bone-RADS. These concepts and frequently asked questions were emphasized during the training phase, and the readers were allowed to review them during the formal evaluation.

**Concept or question**	**Explanation**
The scope of Bone-RADS	A major goal of the panel when creating these algorithms was to be certain that lesions designated Bone-RADS1 are truly benign processes that require no additional workup. While we agree that excessive imaging can be harmful, we want to again emphasize that these flowcharts should never override expert opinion. They can serve as a reference point if there is uncertainty. If an MSK radiologist's opinion in a certain situation deviates from the flowchart in a certain case, then the chart should defer to the expert opinion. It is impossible for any schematic or flowchart to address all permutations and situations. Also, we feel that dismissing a malignant lesion has greater consequences than over-imaging a benign lesion; therefore, we have erred on the side of not ignoring Bone-RADS4 lesions.
Bone-RADS-1	Likely benign, leave alone
Bone-RADS-2	Incomplete assessed on imaging, perform different imaging modality
Bone-RADS-3	Intermediate, perform follow-up imaging (6, 6, 12 months)
Bone-RADS-4	Suspicious for malignancy or need for treatment, biopsy and/or oncologic referral
What is incidental?	The Oxford English Dictionary provides one definition of "incidental" as "occurring by chance in connection with something else." The term "incidental" has become common lexicon in radiology, and we are defining it in this article as "a lesion detected on an imaging study performed for an unrelated reason." An incidental lesion can be definitively irrelevant, of uncertain clinical significance requiring further workup, or concerning requiring treatment, such as a malignancy or infection.
Why only adults?	The Bone-RADS is to present algorithms for the diagnostic management of solitary bone lesions incidentally encountered on CT and MRI in adults. Pediatric patients have a unique set of lesions and a unique appearance of the bone and bone marrow, and therefore are not specifically addressed.
Why only solitary?	We limited our scope to solitary lesions because patients with multiple lesions often have metastatic disease or systemic conditions that typically require biopsy and/or oncologic referral.
Lucent lesions on CT	Lucent lesions are commonly encountered; however, a well-defined quantitative description has not been established. For the purposes of this paper, a "lucent" lesion is

	defined as a lesion that replaces and has lower attenuation than normal trabecular bone, resulting in a hypodense CT appearance. A lesion is defined as lucent if greater than 90% of the volume of the lesion qualifies as lucent and should be evaluated using the "lucent lesion" flowchart. Specially, fat can have an attenuation between − 120 and − 30 HU; non-fatty lucent lesions have HU values between 0 HU and 200 HU; osteoporotic trabecular bone typically is around 120 HU and normal trabecular bone up to 200 HU; gout tophus can be around 160 HU; "ground glass" attenuation of fibrous dysplasia can have a wide range but is typically > 100 HU but well less than 885 HU.
Sclerotic and mixed density lesions on CT	A "dense" sclerotic lesion was defined one where ≥ 50% of its volume is denser than the surrounding normal trabecular bone and "mixed" if the lesion does not reach this threshold. A "mixed" was defined when it has equivalent or near equivalent amount of sclerosis and lucency (1:1 ratio). We have grouped sclerotic and mixed density lesions together in a single flow- chart. Any lesion that does not fit the above definition of a "lucent" lesion should be evaluated as a sclerotic/mixed density lesion.
T1 hyperintense lesions on MRI	T1 hyperintense bone lesions are defined as lesions that demonstrate T1 signal that is visually higher signal intensity than the T1 signal of adjacent skeletal muscle or intervertebral disc
T1 isointense/hypointense lesions on MRI	T1 isointense/hypointense bone lesions are defined as lesions that demonstrate T1 signal that is the same or lower than the T1 signal of adjacent skeletal muscle or intervertebral disc.
T2 hypointense lesions on MRI	T2 hypointense is used to describe lesions with little to no free water that are similar in T2 signal to air or cortical bone, skeletal muscle or fat which has been adequately fat suppresses.
T2 hyperintense lesions on MRI	T2 hyperintense is used to describe lesions that have very high signal similar to fluid (joint effusion, bladder, CSF), including enchondromas and simple bone cysts.
T2 intermediate lesions on MRI	Intermediate T2 signal is less hyperintense than fluid but clearly more hyperintense than skeletal muscle or suppressed fat signal.
Extra MRI assessment	We incorporate chemical shift imaging to identify microscopic fat within the lesion and post-contrast imaging to distinguish cyst-like lesions from solid lesions

To allow the use of the Bone-RADS algorithms, we studied the CT assessment of concerning features, characteristic CT lucent lesions, assessment of lesion density with ROI, T1 hyperintense hemorrhagic lesions can mask underlying lesions, MRI assessment of concerning features, using the example figures from the white paper. The uncertainties were solved by discussion with a multidisciplinary group. The readers were allowed to review them during the formal evaluation.

Finally, the readers were tested by using ten sample lesions from the white paper. When the readers assess the lesions, the figure of the patients and their clinical history were provided, while the readers were blinded to the lesion assessment process, the final diagnosis and discussion section. The readers independently rated the lesions and compared their answers with the final diagnosis from the white paper. If they were the same, the readers were allowed to read the lesion assessment process, the final diagnosis and discussion section to strengthen their comprehension of Bone-RADS. If they were different, the readers were allowed to re-rate the Bone-RADS for the lesion until the answer was correct, then the readers read the lesion assessment process, the final diagnosis and discussion section, and discussed their wrong answers with the multidisciplinary group. Although the current study did not involve the lesions on MRI, the related definitions were provided to completely present the Bone-RADS. Since the current study was on CT only, we emphasized the CT cases during the training phase. The uncertainties were solved by discussion with a multidisciplinary group. The readers were allowed to review them during the formal evaluation.

3. Formal evaluation of Bone-RADS

We prepared a Power Point document (Office 365; Microsoft) to present the images, and an Excel document (Office 365; Microsoft) for patient characteristics. For the cases from center A, although it is possible to allow radiologists view the images through the picture archiving and communication system, it reveals the source of the cases. Therefore, we present the key images to the readers via a Power Point document, in accordance to the cases from other databases. For the cases from center B and center C, it is impossible to view the images through the picture archiving and communication system. Therefore, we present the key images to the readers via a Power Point document. For the Eurorad and Radiopeadia databases, it is impossible to view the specific images blinded to the final diagnosis. Therefore, we present the key images to the readers via a Power Point document. The Power Point document was prepared by two musculoskeletal radiologists with 6 and 7 years of experience (JYZ and YX). This procedure did not allow the readers to view all the images or adjust the images at will as they did in daily routine via the picture archiving and communication system. However, similar setting is common in radiological presentations, articles, and cases.

During formal evaluation, gender, age, and clinical history (presence of pain and malignancy history if possible) of each patient were provided to the readers but blinded to the final diagnosis or the data source. The gender, age, and clinical

history of each patient/lesion was provided to the readers via an Excel document. The Excel document was prepared by two musculoskeletal radiologists with 6 and 7 years of experience (JYZ and YX). After images reading, the readers were asked to record their Bone-RADS rating for each lesion in the Excel document. All the readers independently evaluated the images and rated the Bone-RADS category with no time restrictions, and was allowed to revise their rating until the submission of the Excel document. However, they were told not to discuss the cases with each other.

Supplementary Table S1 Subgroup analysis of diagnostic performance

	TP	FP	FN	TN	Sensitivity	Specificity	Accuracy	DOR
Overall (N = 328)								
Reader 1	198	36	25	69	88.8%	65.7%	81.4%	15.18
Reader 2	203	24	20	81	91.0%	77.1%	86.6%	34.26
Reader 3	203	49	20	56	91.0%	53.3%	79.0%	11.60
Reader 4	211	60	12	45	94.6%	42.9%	78.0%	13.19
Gender								
Male (N = 188)								
Reader 1	120	15	12	41	90.9%	73.2%	85.6%	27.33
Reader 2	122	8	10	48	92.4%	85.7%	90.4%	73.20
Reader 3	122	25	10	31	92.4%	55.4%	81.4%	15.13
Reader 4	128	28	4	28	97.0%	50.0%	83.0%	32.00
Female (N = 140)								
Reader 1	78	21	13	28	85.7%	57.1%	75.7%	8.00
Reader 2	81	16	10	33	89.0%	67.3%	81.4%	16.71
Reader 3	81	24	10	25	89.0%	51.0%	75.7%	8.44
Reader 4	83	32	8	17	91.2%	34.7%	71.4%	5.51
Age								
Age <=45 (N = 168)								
Reader 1	105	18	13	32	89.0%	64.0%	81.5%	14.36
Reader 2	106	16	12	34	89.8%	68.0%	83.3%	18.77
Reader 3	106	25	12	25	89.8%	50.0%	78.0%	8.83
Reader 4	113	32	5	18	95.8%	36.0%	78.0%	12.71
Age >-45 (N = 160)								
Reader 1	93	18	12	37	88.6%	67.3%	81.3%	15.93
Reader 2	97	8	8	47	92.4%	85.5%	90.0%	71.23
Reader 3	97	24	8	31	92.4%	56.4%	80.0%	15.66
Reader 4	98	28	7	27	93.3%	49.1%	78.1%	13.50
Data source								
Local database (N = 178)								
Reader 1	109	15	20	34	84.5%	69.4%	80.3%	12.35
Reader 2	117	13	12	36	90.7%	73.5%	86.0%	27.00
Reader 3	118	25	11	24	91.5%	49.0%	79.8%	10.30
Reader 4	122	31	7	18	94.6%	36.7%	78.7%	10.12
Public database (N = 150)								
Reader 1	89	21	5	35	94.7%	62.5%	82.7%	29.67
Reader 2	86	11	8	45	91.5%	80.4%	87.3%	43.98
Reader 3	85	24	9	32	90.4%	57.1%	78.0%	12.59
Reader 4	89	29	5	27	94.7%	48.2%	77.3%	16.57
CT attenuation								
Lucent density (N = 137)								
Reader 1	101	11	10	15	91.0%	57.7%	84.7%	13.77
Reader 2	103	2	8	24	92.8%	92.3%	92.7%	154.50
Reader 3	102	12	9	14	91.9%	53.8%	84.7%	13.22
Reader 4	108	14	3	12	97.3%	46.2%	87.6%	30.86
Sclerotic or mixed density (N = 181)								
Reader 1	97	25	15	54	86.6%	68.4%	79.1%	13.97
Reader 2	100	22	12	57	89.3%	72.2%	82.2%	21.59
Reader 3	101	37	11	42	90.2%	53.2%	74.9%	10.42
Reader 4	103	46	9	33	92.0%	41.8%	71.2%	8.21

TP = true positive, positive cases that diagnosed as Bone-RADS-2, 3, or 4; FP = false positive, negative cases that diagnosed as Bone-RADS-2, 3, or 4; FN = false negative, positive cases that diagnosed as Bone-RADS-1; TN = true negative, negative cases that diagnosed as Bone-RADS-1; DOR = diagnostic odds ratio; Bone-RADS = Bone reporting and data system.

Supplementary Figure S1 Representative Bone-RADS-1 cases

(A) Reperceive Bone-RADS-1 case A

The following information was provided to the readers when evaluation: “Female; 44 years old; Left hip pain for 4 days.” The final diagnosis of “fibrous dysplasia” was reached by histology.

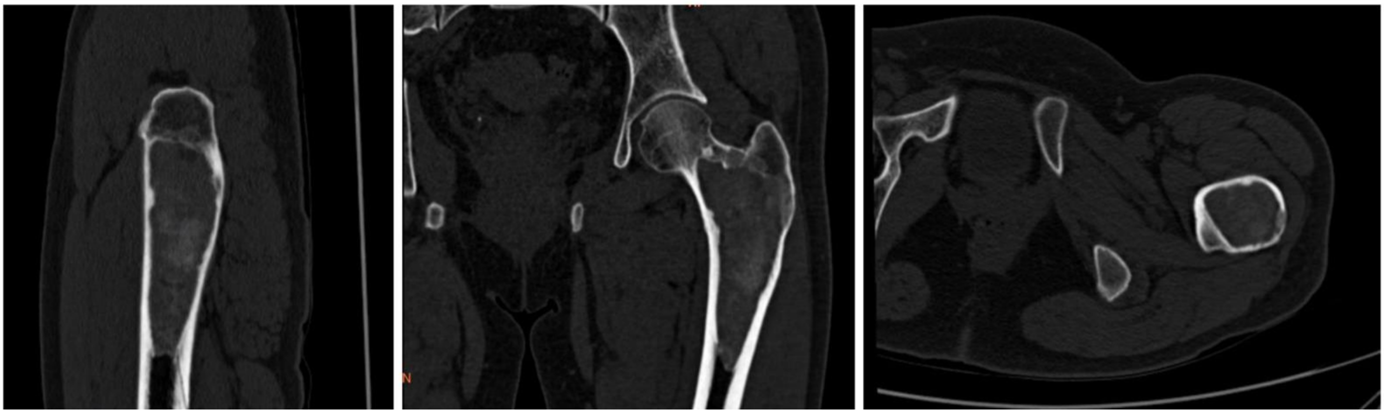

(B) Reperceive Bone-RADS-1 case B

The following information was provided to the readers when evaluation: “Female; 65 years old; Left ankle pain for several months after casual sports.”

The final diagnosis of “subchondral cyst” was reached by the typical appearance and 2 years follow-up.

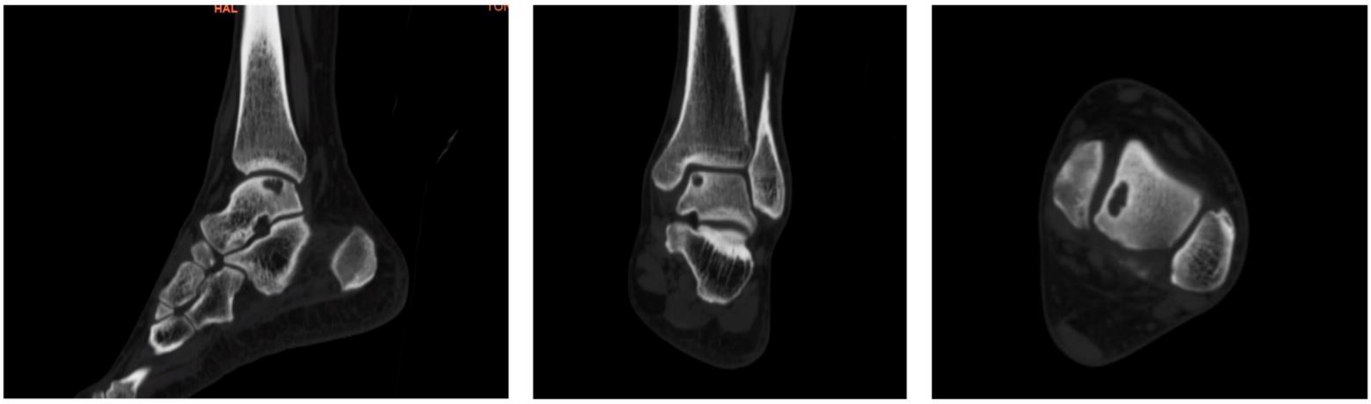

(C) Reperceive Bone-RADS-1 case C

The following information was provided to the readers when evaluation: “Male; 69 years old; Right shoulder injury; incidental bone lesion identified at the head of humerus.”

The final diagnosis of “enchondroma, <5 cm” was reached by the typical appearance and 2 years follow-up. However, the fracture did need further management.

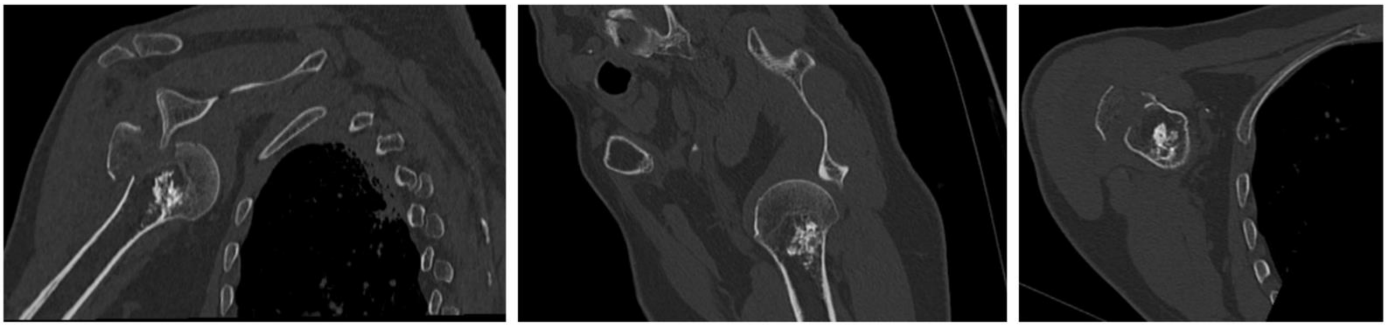

(D) Reperceive Bone-RADS-1 case D

The following information was provided to the readers when evaluation: “Female; 25 years old; Right hip pain for 3 days.” The final diagnosis of “fibrous dysplasia” was reached by the typical appearance and 2 years follow-up.

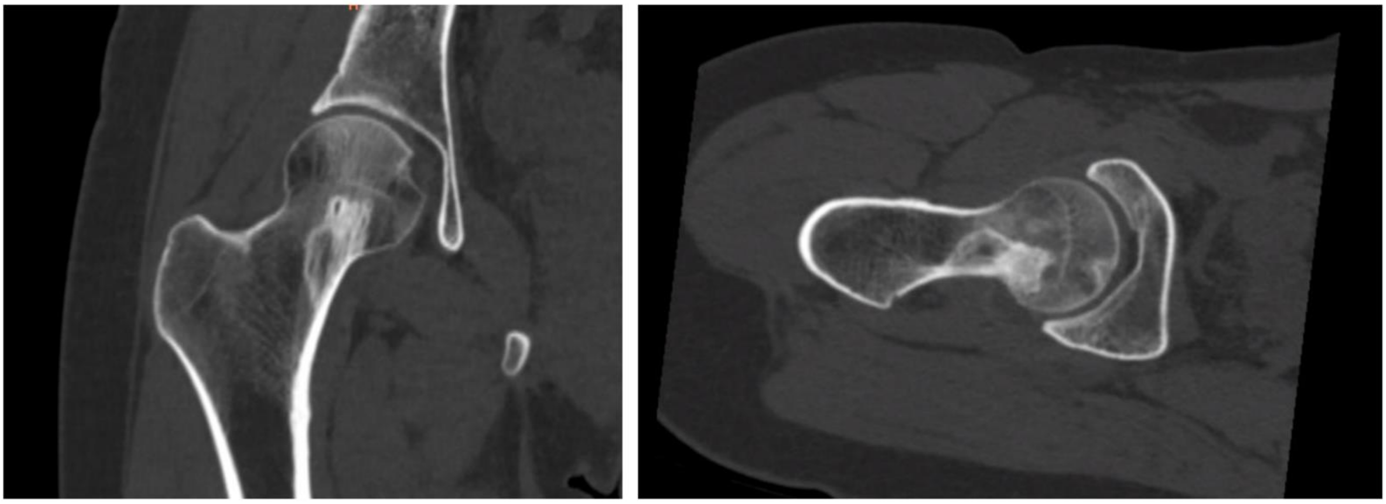

Supplementary Figure S2 Representative Bone-RADS-4 cases

(A) Reperceive Bone-RADS-4 case A

The following information was provided to the readers when evaluation: "Female; 56 years old; Mass of chest wall for 6 months"

The final diagnosis of "chondrosarcoma" was reached by histology.

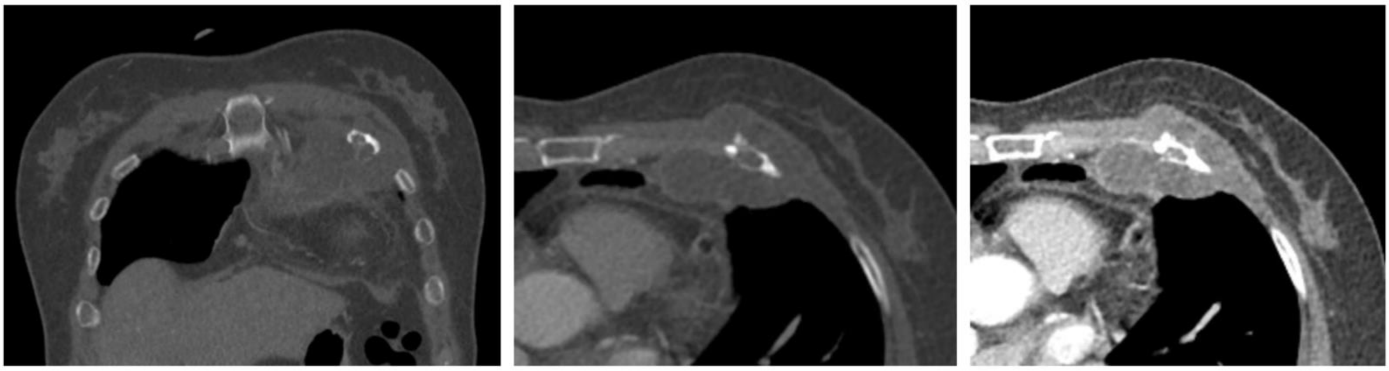

(B) Reperceive Bone-RADS-4 case B

The following information was provided to the readers when evaluation: "Female; 53 years old; Right knee soreness for more than 2 months."

The final diagnosis of "giant cell tumor of bone" was reached by histology.

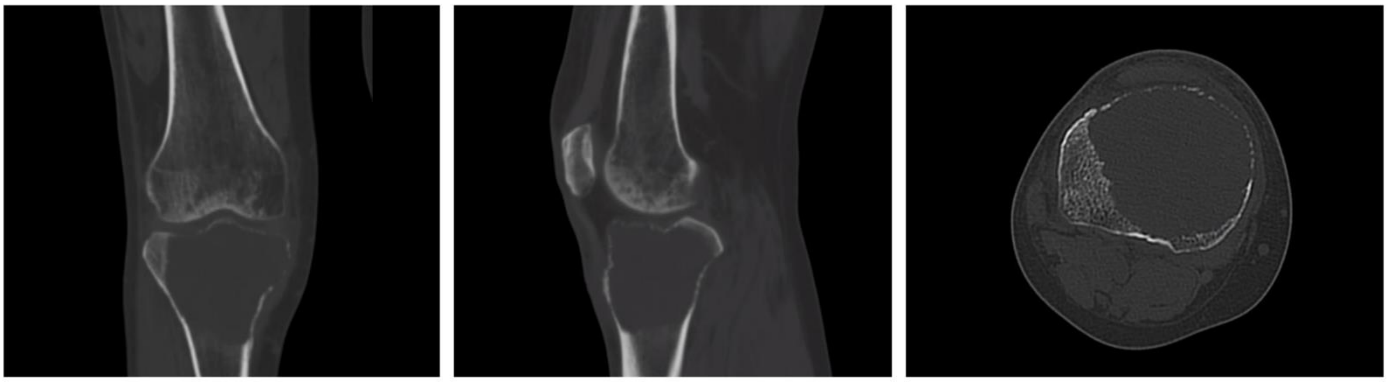

(C) Reperceive Bone-RADS-4 case C

The following information was provided to the readers when evaluation: "Male; 63 years old; History of lung cancer; Right hip pain for 20 days."

The final diagnosis of "bone metastasis" was reached by the typical appearance, and clinical history.

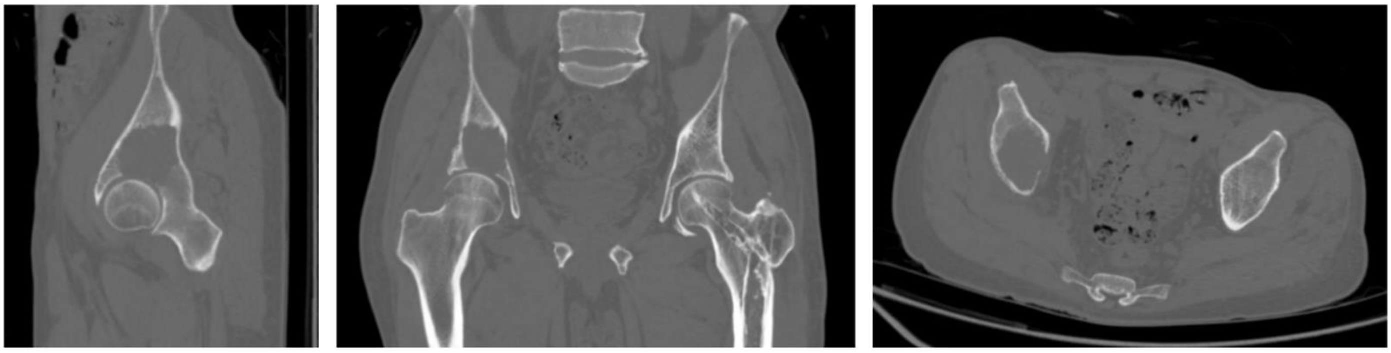

(D) Reperceive Bone-RADS-4 case D

The following information was provided to the readers when evaluation: "Female; 55 years old; History of lymphoma for 2 years; Low back pain for 3 months."

The final diagnosis of "bone metastasis" was reached by the typical appearance, and clinical history.

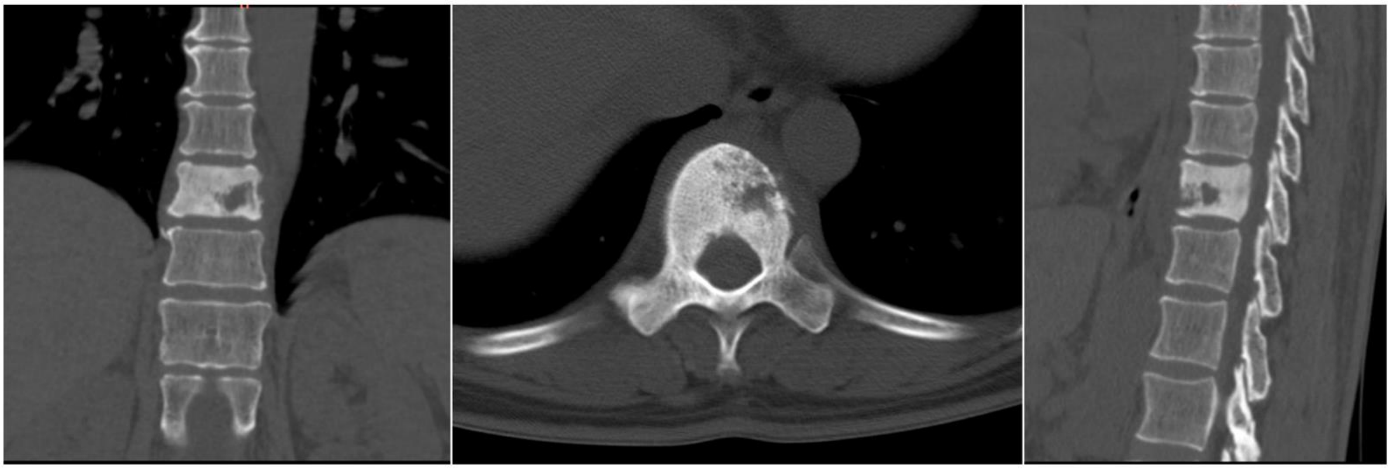

Insights Imaging (2025) Xing Y, Ding DF, Dai S, et al.
